# Problematic khat use as a possible risk factor for harmful use of other psychoactive substances: a mixed method study in Ethiopia

**DOI:** 10.1186/s13011-017-0132-3

**Published:** 2017-11-02

**Authors:** Awoke Mihretu, Solomon Teferra, Abebaw Fekadu

**Affiliations:** 1 Department of Clinical Psychology, Amanuel Mental Specialized Hospital, Addis Ababa, Ethiopia; 2 Department of Psychiatry, School of Medicine, College of Health Sciences, Addis Ababa University, Addis Ababa, Ethiopia; 3 Department of Psychological Medicine, Centre for Affective Disorders, King’s College London, London, UK; 4 Harvard T.H. Chan School of public Health, Boston, USA

**Keywords:** Problematic khat use, Risk factor, Harmful alcohol drinking

## Abstract

**Background:**

Substance use disorders along with neuropsychiatric disorders contributed about 14% of the global burden of disease. Harmful alcohol use, is a known contributor for many harms (accidents, suicide, violence, and complication of other psychiatric and medical disorders). In the Western countries, alcohol and nicotine are gateway drugs to cannabis use, and cannabis use is a risk factor for other illicit drugs such as cocaine and heroin. Khat use is another psychoactive substance which is common in East African and Arabian Peninsula. But there is a knowledge gap regarding the position of khat use or problematic khat use in sequential progression of different psychoactive substances. Therefore, we aimed to understand and investigate the relationship of problematic khat use and other psychoactive substances in Ethiopia.

**Methods:**

Exploratory mixed methods study was employed. Quantitative cross sectional survey was done among 102 khat users, and 4 focus group discussions and 11 in-depth interviews were conducted to understand the pathways between khat use and other psychoactive substances use in 2014. Non random sampling (purposive and snowballing) was employed for both quantitative and qualitative studies. Khat users from khat cafeterias, shops, and from other open markets of khat in Addis Ababa were invited to participate.

**Result:**

Currently significant majorities of khat users (86.3%) used at least one other psychoactive substance after they started khat use. The prevalence of harmful drinking was 53.9% among khat users. Problematic khat use was a significant predictor of harmful drinking (*p* < 0.05). About one from ten respondents engaged to risky sexual behavior pushed by the effect of khat after chewing.

**Conclusion:**

The proportion of psychoactive substances use especially harmful drinking among khat users was observed higher compared to other cross sectional surveys conducted among general population. In Ethiopia, intervention and policy on harmful alcohol use could consider problematic khat use as one possible risky factor. A rigorous methodology which could test gateway hypothesis of problematic psychoactive substances use could be warranted.

## Background

Substance use disorders along with neuropsychiatric disorders contributed about 14% of the global burden of disease [1]. Although polysubstance use is common in many settings, the path of use varies depending on legal status, availability, and other social factors. Previous studies have documented a reliable pattern for initiating substance use, with many adolescents beginning with nicotine and/or alcohol and progressing to illicit drugs [2, 3]. In another setting, regular or heavy cannabis use was associated with an increased risk of using other illicit drugs [4].

Khat use is another psychoactive substance which is common in East Africa, Arabian Peninsula and immigrants living in the west from these countries [5, 6]. In Ethiopia, the national level prevalence of khat use was estimated to 15% [7]. The highest prevalence (64.9%) was observed from the southwestern part of Ethiopia [8] and the lowest in 4% and 7.8% from the northern part [9, 10]. These studies indicated that Khat use was mainly associated with Muslim religion followers, males, alcohol drinkers and cigarette smokers.

Alcohol use, which is the most prevalent drug in Ethiopia [11], is a known contributor to many harms (accidents, suicide, violence, and complication of other psychiatric and medical disorders) [12]. The current prevalence(less than monthly) of alcohol drinking was 15% for women and 36% for men. Among those who drink alcohol, 16% were problem drinkers [13]. In Ethiopia, both life time and current prevalence of other hard drugs is unknown [14]. Among problematic khat users, recent study indicated that about 29% drank alcohol more than weekly, and about (10%) had a history of cannabis use in their life [15].

Studies also suggested the positive associations between levels of khat dependence and nicotine dependence [16]. Among Somalis who lives in London and khat users, 60% of them smoked cigarettes; 75% of these were men, smoking 5–45 cigarettes per day. Very few (6%) used other drugs like cannabis. Gender was an important effect modifier in patterns and correlates of khat use and tobacco use [17]. Age of onset of khat use was also inversely related to the number of cigarettes smoked during a khat session and with intensity of khat chewing [18]. Smoking of tobacco and/or shisha was also reported as drugs which are more common among problematic khat chewers, and are common activities in places where khat is consumed. Cigarettes are believed to facilitate the stimulation effect of khat and to reduce its bitter taste [15]. In a community based cross sectional survey about 61% of khat chewers smoke cigarettes [19]. But many of the studies loosely address the issue of confounders in the process of psychoactive substance uses. Researchers hypothesized that increased khat use and shifting to problematic khat use could be associated with stressful or traumatic life events. This is explained as a self-medication technique in case of emotional and psychological distress. A couple of studies in Somalia indicated that there was a positive association between the number of traumatic events experienced and the quantity as well as duration of khat used [20, 21]. Therefore, based on the existing literatures recommendation on the relationship among different psychoactive substances and confounding variables (age of onset of khat use and stressful life events), we aimed to investigate the relationship of problematic khat use and other psychoactive substances use.

## Methods

We reported the details of methods and material in the previous publication.

### Study design

Both quantitative and qualitative studies were employed to explore why khat use mainly problematic khat use is a risk factor for harmful use of other psychoactive substances and to determine the proportion of other psychoactive substances of use among khat users. Focus group discussions (FGD) and in-depth- interviews (IDI) were conducted for the qualitative part of the study. Quantitative study was relied on cross sectional survey among khat users.

### Participants and sampling

For the quantitative study, 102 participant were participated in the study. There were selected based on purposive and snow balling sampling techniques. Participants were invited to participate from major outlets of khat (khat shops and khat cafeterias) in Addis Ababa, Ethiopia. Similar sampling technique was employed for the qualitative study. We extracted important themes of the qualitative data from four focus group discussions (FGD) and eleven khat users.

### Data collection methods

Socio demographic information was collected using a structured questionnaire. Problematic khat use was measured by using the adopted version of DSM-5 checklists of stimulant use disorders. List of threatening experiences (LTE) and fast alcohol screening tool were used to assess threatening life experiences and harmful drinking respectively. Other psychoactive substances were collected using structured questionnaire. All of the instruments have been used in Ethiopia and had acceptable psychometric properties [22, 23].

### Data analysis

We analyzed the translations of FGD and IDI using thematic method of analysis. The principal investigator did the data collection, transcription and translation, but themes were confirmed through discussion with all of the authors.

We employed descriptive statistics such as frequency and percentage. Chi- square test was to analyze the statistical association between problematic khat use and reasons for using khat. Path analysis were employed to examine the relationship of predictors to problematic khat use and its association with harmful drinking. Statistical package for social science (SPSS v.23) and Analysis of movement structure (AMOS v.23) software was used for the descriptive statistics and path analysis.

### Ethical consideration

Ethical approval was secured from ethics committee of the school of Psychology, Addis Ababa University. Autonomy, informed consent, harm and benefit were maintained throughout the study.

## Result

The socio demographic characteristics of respondents were discussed elsewhere. We collected data on 102 khat users (age range from 19 to 65) in Addis Ababa. Majority of the respondents were males 80(78.4%). Nearly half of the participants (49.0%) had started chewing khat before the age of 10. Majority of the participants (*n* = 35; 34.3%) chewed khat on a daily basis. For the qualitative study, 7 women and 27 men participated in the FGD and 13 participants were also participated in the in-depth interview. The majority of the participants’ age was between 32 and 40 years. Most (*n* = 26) had at least high school diploma (Table 1).

**Table 1:** Background information of participants (*N* = 102)

Characteristics	Frequency	Percent
Age		
15–24	24	23.5
25–34	45	44.1
35 and above	31	30.4
Sex		
Male	80	78.4
Female	22	21.6
Religion		
Orthodox	66	64.7
Muslim	24	23.5
Protestant	10	9.8
Catholic	2	2
Marital status		
Single	71	69.6
Married	24	23.5
Divorced	6	5.9
Other*	1	1
Living arrangement		
With parents or other Relatives	60	58.8
With partner	15	14.7
Alone	26	25.5
Other**	1	1
Wealth(perceived wealth status compared to neighbors)		
Low	38	37.3
Medium	54	52.9
High	8	7.8
Ethnicity		
Oromo	17	16.7
Amara	41	40.2
Guragie	18	17.6
Tigria	13	12.7
Others***	8	8.8
Employment		
Private business	20	19.6
Student	12	11.8
Employed	48	47.1
Daily laborer	6	5.9
Jobless	5	4.9
Petty trade	9	8.8
Other****	2	2
Total	102	100

*** welayta, selti, ****commercial sex workers ** quit from home due to khat chewing behavior *windowed

### Reasons for khat use

Respondents start chewing khat for different reasons. The Majority (about half) of the respondents did for functional (academic and work) (*n* = 41; 40%) and/or an alternative to spend spare time (*n* = 45; 44%). About one third of the total respondents (*n* = 32; 31.4%) maintain their khat use behavior because they believe they are “addicted” (Table 2). In order to investigate the statistical association between reasons for using khat and being problematic khat use, chi-square test for independence was run out. Respondents who use khat being “addicted” was statistically and significantly associated with problematic khat use. Problematic khat use was also associated with using khat for self-treatment from emotional distress, *p* < 0.05.

**Table 2:** Reasons for khat use and being problematic khat use

Reasons for khat use	*F*	Percent	χ2 value	*sig. (2-tailed) at 1 df*
Religious	9	8.8	0.00	*1*
Culture	7	6.9	0.001	0.98
To drink	8	7.8	3.5	0.06
For functional purpose	41	40.	0.24	0.88
To spend spare time	45	44	0.29	0.62
Being khat “addict”	32	31.4	7.3	0.007*
Conditioned by the event at khat cafeteria/*meqamia bête*	12	11.8	0.9	0.34
Self-treatment from emotional distress	28	27.4	4.2	0.04*
Other**	4	3.9		

*p < 0.05 **to be calm, for its euphoric effect

The qualitative data also had explanation for why people engage and stay in khat chewing behavior.

*I personally start to chew because of the influence of others. A wealthy man asked me to buy khat for him and he highly influenced me to chew with him. Through time, I found the stimulating effect of khat interesting and I continued chewing, this is how I started chewing. Now I maintain my behavior to treat the distressing experiences I feel when I didn’t chew.* (IDI#7, age 68)

### Path way of psychoactive substances use among khat users

**Fig. 1:**
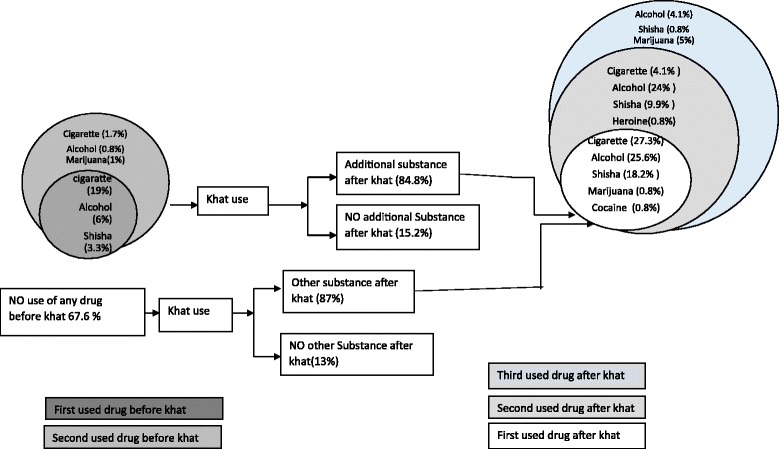
Suggested that the local model of problematic substance use could be 1) khat use; 2) cigarette use; 3) harmful drinking; 4). Marijuana use and other hard drugs

The path of respondents’ use of psychoactive substances before and after they starting chewing khat is indicated in the diagram below (Fig. 1). Among the respondents 67.6% (*n* = 69) didn’t have used any other psychoactive substances other than khat in their life time but after starting khat use, 87% (*n* = 88) of the respondents use other psychoactive substances. Using fast alcohol screening test the prevalence of harmful drinking was 53.9% (*n* = 55). One fifth of the respondents (19%, *n* = 23) started cigarette smoking before using any other psychoactive substances including khat. The use of hard drugs and harmful drinking is common later after respondents become khat users.

From the qualitative study, respondents discussed how and why khat use is associated with use of other psychoactive substances. They confirmed that it was common to see smoking cigarette and *shisha*/water pipe while chewing khat and drinking alcohol after chewing khat.

Smoke cigarette and shisha/water pipe, and drinking coffee were common during chewing khat than other times which are important to intensify the stimulation from khat, in khat cafeterias and done being in group. The major reason for respondents to drinking alcohol is, to break the stimulating effect of khat so that the alcohol will be helpful to induce sleep since insomnia is the typical feature of khat and for self-treatment from khat induced distressing experiences. Khat chewers blamed khat for its *catalyst* nature. There are also other techniques practiced by respondents to break the stimulating*/mirqanna* of khat in addition to drinking (Table 3). Here are two respondents’ experiences.

*Cigarette and khat have sometimes relationship but not always. This is because I smoke without chewing but in another time I smoke an increased amount while I only chew khat to increase the stimulation. They have also a relationship because while I didn’t chew I smoke or drink coffee in an increased amount than other times. I drink alcohol rarely just during holidays* (IDI#6, age 43).

**Table 3:** Different techniques used by khat users to break the stimulating*/mirqanna* effect of khat use

Techniques used to break the after effect of khat/*mirqanna*	*f*	Percent
Drinking alcohol	57	55.9
Risky sexual engagement	14	13.7
Drinking milk	13	12.7
Personal techniques(sport, long walk, washing body with cold water)	7	6.8
Suffer and doing nothing	4	3.9
No distress	30	29.4

Another respondent;

*I used cigarette and shisha in addition to khat. I use cigarette all the time but I smoke more while I chew. I used shisha only during chewing. I drink every time I chew because I get relief from confusion and internal fear just after I reverse the over stimulation/mirqanna of khat. After I start chewing, I am alcohol addict and I do sex regularly with sex workers. Really, khat is akatari (catalyst) (IDI# 4, age 36).*

### Path analysis for the process of problematic psychoactive substances uses

#### Model fitting assessment

Chi-square test was non-significant; *p* > 0.05. Other indices such as RMSEA (root mean square error of approximation) which is the average of the residuals between the observed correlation/covariance from the sample and the expected model estimated from the population is 0.053 (RMSEA = less than .08 is good fit(25). CFI (comparative fit index) was also approximate to the recommended value (> 0.95). Considering other assumptions of path analysis and model fitting the following model is the final selected and approved model [24].

**Fig. 2:**
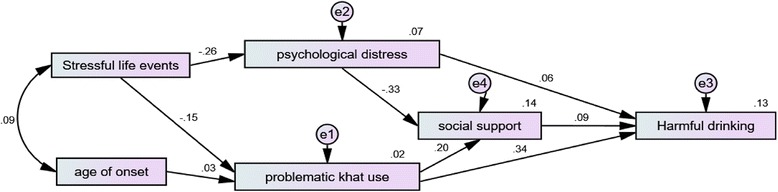
The path diagram with standardized path coefficient of exogenous and endogenous variables for harmful alcohol drinking

The standardized and total effect of variables is indicated below (Fig. 2). Problemtic khat use (unstandardized path coefficient) accounted 56% of the variace of harmful alcohol drinking. The standardized path coefficient revealed that 34% of the variance of harmful alcohol drinking was explained by problemtic khat use. Therefore, problemtic khat use was a statistically significant predictor of harmful alcohol drinking both directly and througth social support as a mediating variable(*p* < 0.05). There was also an inverse statistically significant association between psychological distress and social support(the unstandardized and standardized path coefficient was ***−.***22 and **−**.33 respectively; *p* < 0.05) (Fig. 2).

## Discussion

Khat use has a similar function (relaxation, enhance activity, decreased boredom, and avoid unpleasant feelings with not using the drug) with other psychoactive substances like cannabis and alcohol use [25]. There is no significant difference in terms of reasons of using khat across different settings like Saudi Arabia [26], Yemen [27] and different regions in Ethiopia. Many studies that people use khat to increase concentration and performance (praying, work, and academics), to spent ample time, and pressured by peers. Male sex and Muslim religion were frequently reported associated factors [7, 9, 28, 29]. Despite the endorsement a few respondents by the current study, khat use has been also embedded to culture and practised as a social custom in Ethiopia, Yemen, Somalia and Kenya [30], and among immigrants of these countries live in European and America. The current study could be different from the previous studies by reporting a significant number of khat users (31.4%) use khat because they perceive they are *addicted* to khat. This difference was observed because the current study focused on khat users mostly problematic users than general population.

The findings of the study suggested increased number of people had harmful alcohol drinking behavior among khat users than the reports from the general community [13, 31]. This could be the difference in setting and type of respondents. The current study targets on adults in city where problematic khat users are common. Problematic khat use was statistically significantly associated with harmful alcohol drinking adjusting for age of onset of khat use, stressful life experiences, psychological distress and social support. There are also other similar studies which suggested that khat use might be a gate way for other psychoactive substances (alcohol and cigarette) [28, 32, 33]. The current study was consistent with this and suggested problematic khat use, than khat use per se could be a risk factor for harmful use of other psychoactive substances especially harmful alcohol use. Cigarette was mainly used to maximize the stimulation power of khat (during chewing) and alcohol is practiced to break the aftereffect. This leads the individual to be harmful use of polysubstance uses. Previously we had reported that: *if you didn’t chew you will not be forced to smoke, drink alcohol or to engage in risky sexual intercourse* *.* So, Khat is an antidote for alcohol and can counter drunkenness (an idea bound up with its symbolic opposition to alcohol with religious beliefs of some East Africa and Arab Muslims) [34] and drinking milk could be the option for these groups of people.

As endorsed from the result section, the local model of problematic substance use could be 1) khat use; 2) cigarette use; 3) harmful drinking; 4). Marijuana use and other hard drugs. Problematic khat use could overlaps with khat use and other psychoactive substances uses. This looks comparable to Kandel’s (1975) model in the western culture but khat use is a local drug which has a cultural use component and more morally acceptable than other drugs [2]. Marijuana is still considered as very harmful, immoral and hard drug in local context and it has a recent history of use among users.

There are also a number of participants (13%) who engage in risky sexual behavior pushed by the stimulating/*mirqanna* of khat. Other studies had also confirmed the positive association between khat use and risk factors (HIV infection and other psychoactive substances uses. Especially, daily Khat intake was associated with unprotected sex and multiple sexual practices and in turn with HIV infection [35, 36]. Looking for a sexual activity as an option to break the stimulation of khat is also observed in another setting, Australia [34]. The after effect of khat use to sexual desire is controversial. But it could be because of release to neurotransmitters norepinephrine and dopamine which increased sympathetic nervous system (SNS) activity, and this will have a role in sexual desire [35]. Another explanation for the significant association between khat use and alcohol drinking is that many khat chewers prefer drinking alcohol to break the after effect-*mirqana, after* khat session mostly at noon*.* Despite reporting interesting exploration and employing advanced statistical models to understand and examining the risk of problematic khat use for other risky behaviors, and psychoactive substances use, the study was limited to include other important explanatory variables(years of exposure, effect of availability, access, exposure, and economics of all psychoactive substances) in the model. Harmful use of other psychoactive substances (cannabis, cocaine, and heroin) were not rigorously measured and included in the path analysis model. Because of these two limitations and the nature of the research design, cross sectional survey, the study couldn’t establish which drug is a gate way for the others in Ethiopian setting.

## Conclusion

In general, the study had significant implication on how problematic khat use is potential risk factor for other psychoactive substance use (mainly harmful drinking) and other physical health harms associated with risky sexual behavior. Proportion of other psychoactive substance use especially harmful alcohol drinking was higher compared to the general population. Problematic khat use was also the main predictor of harmful drinking. Generally, conceptualization, intervention and policy on substance use disorders could give prior emphasis for problematic khat use.

## Abbreviations

AMOS: Analysis of movement structure
DSM: Diagnostic statistical power
FAST: Fast alcohol screening test
FGD: Focus group discussion
IDI: In-depth interview
LTE: List of threatening experiences
OSS: Oslo social support
SPSS: Statistical package for social science
